# A Facile One-Pot Synthesis of α-Bromo-α,β-unsaturated Esters from Alcohols

**DOI:** 10.3390/molecules15053276

**Published:** 2010-05-04

**Authors:** Usama Karama, Zeid Al-Othman, Abdullah Al-Majid, Abdulrahman Almansour

**Affiliations:** Chemistry Department, College of Science, King Saud University, P.O. Box 2455 Riyadh 11451, Saudi Arabia

**Keywords:** one-pot synthesis, bromoylides, oxidation, α–bromo-α,β-unsaturated esters

## Abstract

Treatment of *N*-bromosuccinimide (NBS) with (carboethoxymethylene) triphenylphosphorane (**1**) in CH_2_Cl_2_ followed by the addition of an alcohol in the presence of manganese dioxide under ultrasonic irradiation constitutes a stereoselective one-pot procedure for the preparation of *Z*-configured α–bromo-α,β-unsaturated esters in good to excellent yield.

## 1. Introduction

The Wittig reaction is one of the most powerful and attractive methods for the construction of various alkenes [1]. Nicolaou and his coworkers [2] have highlighted the utility of the Wittig olefination and related reactions in natural products synthesis both in the research laboratory and industrial settings. α–Halo-α,β-unsaturated esters are useful intermediates in organic synthesis [3]. For example, the synthesis of α–trifluoromethyl-α,β-unsaturated esters was based on trifluoromethylation of α–iodo-α,β-unsaturated esters [4] as well α-bromo-α,β-unsaturated esters [5]. They are usually prepared by the Wittig reaction of aldehydes with haloylides [6,7] and condensation of aldehydes with halo-phosphonates in the presence of a base [8]. Only aldehydes have been used as substrates in this typical method. Taylor [9] described the development of an *in situ* alcohol oxidation-Wittig reaction using (carboethoxymethylene)triphenylphosphorane and manganese dioxide to produce α,β-unsaturated esters. This method avoids the most common problems associated with the handling of intermediate aldehydes, which are often difficult to isolate due to their volatility, toxicity, penchant to polymerize or facile hydration [9], and unsuitable for use as substrates.

In this paper we describe a one-pot methodology for the synthesis of (*Z*)-α–bromo-α,β-unsaturated esters using the above procedure.

## 2. Results and Discussion

**Scheme 1:**
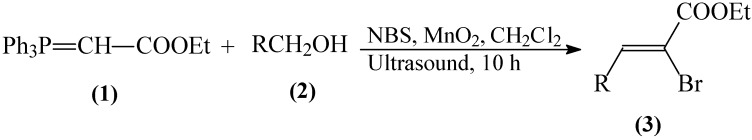
Tandem halogenation-oxidation-Wittig reaction.

Due to our continuing interest in one-pot procedures [10,11], which convert multi-step reactions into economically and environmentally favored one-pot processes, we decided to combine the halogenation of an ylide, the oxidation of an alcohol with the cheap reagent MnO_2_ as a oxidant and a subsequent Wittig reaction into a one-step procedure. We utilized (carboethoxymethylene)triphenyl-phosphorane (1) as starting material to react with activated alcohol in the presence of *N*-bromo-succinimide and manganese dioxide and found that the tandem reaction of halogenation-oxidation-Wittig reaction promoted by ultrasound wave took place readily to form the desired product 3 (Scheme 1). 

As shown in Table 1 the one-pot sequential halogenations-oxidation-Wittig reaction of reactive alcohol such as aromatic, allylic, and propargylic alcohols furnished the corresponding α-bromo-α,β-unsaturated esters in good yield and very high stereoselectivity. The yield for unreactive alcohols like alkanols was rather low (entry 7). The *E* and *Z* isomers were not separated in any of the reactions, but the *Z*:*E* ratios were readily determined by ^1^H-NMR spectroscopy as the vinylic protons of the *Z* isomers were downfield of those of the *E* isomers. This assignment was confirmed by NOE analysis of the allylic alcohol derived from the corresponding esters by DIBAL reduction.

**Table 1:** One-pot synthesis of (*Z*)-α-Iodo-α,β-unsaturated esters.

Entry^a^	R	Product	Yield (%) ^b^	Z:E ^c^
**1**	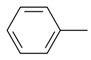	**3a**	85	94:6
**2**	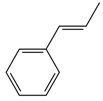	**3b**	81	95:5
**3**	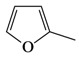	**3c**	91	89:11
**4**	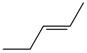	**3d**	90	100:0
**5**	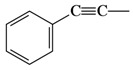	**3e**	86	92:8
**6**	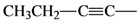	**3f**	87	76:24
**7**	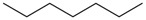	**3g**	21	90:10

^a ^One equiv. of alcohol, 1.3 equiv. of (1), 1.4 equiv. of NBS and 10 equiv. of MnO_2_ were used and reaction mixture was sonicated for 10 h; ^b^ Yields were based on alcohols; ^c^ This ratio was determined by ^1^H-NMR spectroscopy.

## 3. Experimental

### 3.1. General

IR spectra were recorded on a Perkin-Elmer 883 spectrophotometer and are expressed as ν in cm^-1^ NMR spectra were recorded on JEOL ECP 400 (400 MHz) in CDCl_3_ and chemical shifts are expressed as δ in ppm, and coupling constants (J) were given in Hertz. MS spectra were obtained using EI at 70 eV. The ultrasonic reactions were carried out using a Sonerex 200 operating at 50 W power and a frequency of 35 kHz.

### 3.2. General procedure for the synthesis of (Z)-α–bromo-α,β-unsaturated esters

Activated manganese dioxide (purchased from Aldrich, 10 mmol) was added to a solution of (carboethoxymethylene)triphenylphosphorane (1.3 mmol), *N*-bromosuccinimide (1.4 mmol) and alcohol (1 mmol) in CH_2_Cl_2_ (12 mL) and the mixture was sonicated for 10 hours. The manganese dioxide was removed by filtration through Celite, which was washed well with CH_2_Cl_2_, and the combined organic portions were concentrated under vacuum to ca. 1-2 mL, the residue was purified by column chromatography on silica gel (petroleum ether-ethyl acetate 15:1).

*(Z)-Ethyl 2-bromo-3-phenylpropenoate* (3a)[12]: Yellow oil, ^1^H-NMR: δ = 1.38 (t, *J* = 7.3 Hz, 3H); 4.33 (q, *J* = 7.3 Hz, 2H); 7.41-7.45 (m, 3H, arom-H); 7.83-7.85 (m, 2H, arom-H); 8.21 (s, 1H); IR (ν_max_, thin film) 2980, 1724, 1610, 1259, 1037, 765; MS (EI), m/z (%) = 256 (54), 254 [M^+^] (54), 175 (91), 174 (100), 102 (97).

*(2Z, 4E)-Ethyl 2-bromo-5-phenylpenta-2,4-dienoate* (3b) [12]: Yellow oil, ^1^H-NMR: δ = 1.37 (t, *J* = 7.3 Hz, 3H); 4.29 (q, *J* = 7.3 Hz, 2H); 7.04 (d, *J* = 16.1 Hz), 7.13-7.19 (dd, *J* = 10.3 Hz, *J* = 16.1 Hz), 7.32-7.39 (m, 3H, arom-H); 7.51-7.53 (m, 2H, arom-H); 7.81 (d, *J* = 10.3 Hz); IR (ν_max_, thin film) 2980, 1714, 1614, 1583, 1261, 1041, 744; MS (EI), m/z (%) = 282 (26), 282 [M^+^] (27), 155 (47), 129 (100), 102 (25).

*(Z)-Ethyl 3-(furan-2-yl)-2-bromo-3-propenoate* (3c) [12]: Yellow oil, ^1^H-NMR: δ = 1.33 (t, *J* = 7.3 Hz, 3H); 4.30 (q, *J* = 7.3 Hz, 2H); 6.54 (m, 1H), 7.42 (d, *J* = 3 Hz, 1H), 7.57 (d, *J* = 2 Hz, 1H); 8.13 (s, 1H); IR (ν_max_, thin film) 2981, 1716, 1249, 1039, 748; MS (EI), m/z (%) = 246 (39), 244 [M^+^] (38), 165 (58), 137 (100), 92 (30).

*(2Z, 4E)-Ethyl 2-bromohepta-2,4-dienoate* (3d): Yellow oil. ^1^H-NMR: δ = 1.04 (t, *J* = 7.3 Hz, 3H); 1.31(t, *J* = 6.6 Hz, 3H); 2.22 (quint. *J* = 7.3 Hz, 2H); 4.25 (q, *J* = 7.3 Hz, 2H); 6.32-6.44 (m, 2H); 7.63 (d, *J* = 10 Hz, 1H); IR (ν_max_, thin film) 3055, 1726, 1265, 705; MS (EI), m/z (%) = [M^+ ^cannot be detected], 208 (41), 206 (36),106 (39), 104 (39).

*(2Z)-Ethyl 2-bromo-5-phenylpent-2-en-4-ynoate* (3e): Yellow oil. ^1^H-NMR: δ = 1.34 (t, *J* = 7.3 Hz, 3H); 4.33 (q, *J* = 7.3 Hz, 2H); 7.29-7.58 (m, 4H, arom-H); 7.50 (s, 1H); IR (ν_max_, thin film) 2980, 1718, 1265, 738; MS (EI), m/z (%) = 280 (42), 278 [M^+^] (42), 199 (73), 171 (100), 106 (39), 126 (97).

*(Z)-Ethyl 2-bromohept-2-en-4-ynoate* (3f): Yellow oil. ^1^H-NMR: δ = 1.21 (t, *J* = 8 Hz, 3H); 1.30 (t, *J* = 7.3 Hz, 3H); 2.44 (dq, *J* = 7.3 Hz, *J* = 2.2 Hz, 2H), 4.27 (q, *J* = 7.3 Hz, 2H, 7.26 (t, *J* = 2.2 Hz, 1H); IR (ν_max_, thin film) 2998, 1718, 1265, 740; MS (EI), m/z (%) = 232 (49), 230 [M^+^] (51), 186 (40), 151 (96),77 (100).

*(Z)-Ethyl 2-bromonon-2-enoate* (3g) [13]: Yellow oil, ^1^H-NMR: δ = 1.24 (t, *J* = 7.3 Hz, 3H); 1.27-1.32 (m, 11H), 2.33 (q, *J* = 7.3 Hz, 2H), 4.27 (q, *J* = 7.3 Hz, 2H); 7.27 (t, *J* = 7.30 Hz, 1H); IR (ν_max_, thin film) 2926, 1732, 1259, 1041, 802.

## 4. Conclusions

In summary, we have developed an efficient ultrasonic-assisted one-pot general procedure for a domino primary alcohol oxidation-Wittig reaction for the highly stereoselective synthesis of Z-α-bromo-α,β-unsaturated esters **3**. These results demonstrate the value of ultrasonic- assisted chemistry.
